# The Relationship between Chronic Non-Communicable Diseases of Fish Farm Household Members and Production Efficiency: The Case of Ghana

**DOI:** 10.3390/ijerph20054175

**Published:** 2023-02-26

**Authors:** Martinson Ankrah Twumasi, Dennis Asante, Jesse Nuamah Brako, Zhao Ding, Yuansheng Jiang

**Affiliations:** 1 College of Economics, Sichuan Agricultural University, Chengdu 611130, China; 2 College of Medicine & Public Health, Rural and Remote Health, Flinders University, Renmark, SA 5341, Australia; 3 Akim Oda Government Hospital, Akim Oda City P.O. Box 16, Eastern Region, Ghana

**Keywords:** data envelopment analysis, human diseases, aquaculture production efficiency, Ghana

## Abstract

Prior studies explored the production and technical efficiency of fish farms and farmers from the perspectives of factors such as credit access and cooperative membership. We focused on the chronic non-communicable diseases (NCDs) of household members and their quantitative impacts on fish farm production efficiency, based on data of earthen pond fish farms from two regions (Bono East and Ashanti) in Ghana. A data envelopment analysis (DEA) and the IV Tobit technique were employed for the study’s analysis. From the study’s observations, we draw the following conclusions. We found that the NCDs of household members indeed reduce farm production efficiency, and the heterogeneous impact of the NCDs of female members on farm production efficiency was more prominent than that of male members. Insights from this study suggest that the national government should provide farmers with the necessary medical care through the provision of subsidized health insurance, which can facilitate access to healthcare services. Moreover, NGOs and governments should encourage health literacy, i.e., organizing programs aimed at educating farmers on NCDs and their impact on agriculture.

## 1. Introduction

While providing proper nutrition and food security for a growing population worldwide, the aquaculture and fishery sector is alleviating poverty, improving livelihoods, and creating many job avenues for small- and medium-scale producers [1,2]. Despite the global trend, aquaculture in Africa, including Ghana, lags compared to other regions in terms of global fish production [3]. For example, Africa’s estimated net import of fish will be around 5 million tons by 2030, while half of its fish consumption needs will be met through importation by 2050 [4]. The low growth in African aquaculture can be attributed to various factors such as the prevalence of outmoded technology, inadequate fish species, scarcity of quality feeds, inadequate technical resources, financial and human resource constraints, low technical efficiency, inefficient research and development systems, and weak market infrastructure and access [5,6,7]. 

We argue in this study that NCDs and fish farm efficiency or productivity may relate to each other. The World Health Organization (WHO) global report in 2005 showed that alleviating NCDs must be one of the priorities of every nation, because NCDs reduce economic growth [8]. Thus, NCDs may have a detrimental effect on farm efficiency or productivity due to the inadequate and poor quality of the labor supply, high cost of expenditure on NCDs’ treatment, and diversification of productive time to care for the sick [9,10,11,12,13]. We further argue that male and female members with NCDs may affect efficiency differently. The NCDs of male and female household members may exert different effects on the labor supply. Characteristic of most African households, male household heads in Ghana are the breadwinners; therefore, off-farm jobs are predominant among male members [14]. This presupposes a distinct division of labor. Since off-farm jobs are dominated by males, their female counterparts generally engage in homestead farm activities. Thus, male and female members with NCDs may have a disaggregated effect on efficiency. The scenarios given show that there is a potential association between NCDs and efficiency; however, previous studies [6,15,16,17] highlighted the problem of farmers/farms’ inefficiency from the perspectives of other factors rather than household members’ health. Although the World Health Organization [18] defines health as not merely the absence of disease but also individuals’ physical, mental, and social well-being, this present study focuses on the absence of NCDs because of their impact on efficiency and cost. Thus, we analyze the effect of the NCDs of fish farm’s household members on their farm’s production efficiency. Given the significant effect of improving fish farm production efficiency, which enhances productivity, food security, and income, it is imperative to investigate the factors that could improve fish farm production efficiency. A fish farm with all the necessary tools to improve efficiency, which affect output maximization, is likely to not achieve its efficiency target if any of the fish farm’s household members are bombarded with NCDs. This is because farm efficiency, which affects farm productivity, is influenced by the producer’s health status and that of their household [10]. 

Among all the studies assessing the efficiency of fish farmers and fish farms in Ghana [6,19,20] and other countries such as Myanmar, Norway, and Malaysia [21,22,23,24], none have quantitatively addressed the relationship between the household members’ NCDs and the fish farm’s production efficiency. Therefore, this study fills this gap by employing data collected from two regions in Ghana, Ashanti and Bono East. The objectives of the current study are twofold. First, we examine the impact of the household members’ NCDs on fish farm production efficiency. Second, the study analyzes the heterogeneous impact of the household members’ NCDs on farm efficiency by gender composition. The following contributions are made to the extant literature. First, we add to the aquaculture development literature by providing the first quantitative study estimating the household members’ NCDs and fish farms’ efficiency nexus, particularly in Sub-Saharan Africa (SSA). Looking at the detrimental effect of NCDs among farm households and the low development of aquaculture in Africa, this study is timely to suggest solutions to improve agricultural growth. Second, in addition to the NCDs and production efficiency of fish farm household association analysis, this study further analyzes the disaggregated association between the two variables based on gender composition. Since there may be a division of labor, which indicates different roles by gender within a household, the heterogeneous analysis is essential. Finally, we offer insights on how to effectively deal with the endogeneity problems associated with the focal and treatment variable (in this study, the household members’ NCDs) in primary data analysis. We provide the basis for developing targeted policies aimed at enhancing fish production efficiency, which can enhance economic development. 

The rest of the paper is organized as follows. Section 2 discusses the methodology, including a description of the study area, the survey procedures and data, and the empirical models for the analysis. Section 3 and Section 4 present the results and discussions and the conclusion of the study, respectively. 

## 2. Methodology

### 2.1. Data Source

Data for the study’s analysis was gathered from 131 fish farm households from two regions in Ghana between February and April of 2018. A multi-stage sampling technique was employed in the chosen regions (Ashanti and Bono East) because of the higher presence of smallholder fish farms compared to other regions. Within these regions, households with earthen ponds were included in the study because the majority of the fish farms in these regions utilize earthen ponds for production compared with other methods. Utilizing the zonal demarcations developed by the fisheries commissions in respective administrative regions for monitoring and interventions, 70 farmers were sampled from each of the two regions. This process was actively aided by the leaders of various farming associations. However, of the 140 sampled participants, 9 questionnaires were discarded from the final analysis because of missing data and inaccurate responses. 

We used a structured questionnaire to gather our data from the farmers. Local native speakers were recruited to assist us in a face-to-face interview to make the questions understandable to the respondents. To avoid potential ambiguities, we pretested the questionnaire instrument for content validity and reliability through a pilot study. The survey instrument covered information on participants’ socioeconomic and NCDs status; business characteristics, including farm-level inputs and outputs; constraints to fish farming; credit accessibility; off-farm jobs; and other aspects related to the study (Table 1). Standard data preparation and cleaning procedures such as editing and coding for validity, accuracy, and consistency were carried out using SPSS 26 and Stata 14.

### 2.2. Variable Selection and Measurement

This research investigates the effect of household members’ NCDs on the production efficiency of Ghanaian fish farms. Therefore, we had to find the determinant of NCDs of household members and insert it into the outcome equation. Based on de-Graft Aikins [25] and information gathered from the Center for Disease Control and Prevention (CDC) in Ghana, 10 NCDs were selected for this study. The selected chronic diseases include cardiovascular disease, arthritis, hypertension, diabetes, asthma, cancer, chronic headache, chronic pneumonia, chronic kidney, and stroke. The respondents were asked to select from the list or specify if at least one member in the household was suffering from any NCDs. In this study, the respondent’s status was taken as the valid answer, regardless of it being confirmed by a medical practitioner. The reason is that in rural Africa, a lot of people do not go for regular medical checkups, so they may harbor disease or identify and treat them using traditional methods [26]. In addition, household action depends on what the members of the household believe and how they act. Thus, if they believe a member is suffering from NCD, they start treating it either traditionally or using over-the-counter medications. Given this scenario, the independent focal variable, NCDs of household members, is defined as the number of household members with NCDs in a household. Moreover, we defined female and male members with NCDs as the number of female and male members with NCDs in a household, respectively. Household members refer to individuals living together or sharing economic benefits, normally consisting of but not limited to parents, children, and elders. 

To measure the fish farm’s output, the authors’ used the amount of fish, measured in kilograms, produced per hectare within the 12 months prior to the survey. Existing data on peasant agriculture indicates that household outputs can be captured using the above method [6,19].

### 2.3. Empirical Model Specification 

We use a two-staged DEA approach for our study. Initially, the DEA was used to measure the fish farm production efficiency. The Tobit model was later applied to identify sources of inefficiencies in the second stage, because the efficiency scores were within the range between 0 and 1. This technique was employed in a larger strand of the literature to determine efficiency matching scores [27,28,29]. 

DEA approach is a non-parametric method used in establishing efficiency of Decision-Making Units (DMUs), where multiple input variables are used to produce an output [30]. Under DEA, efficiency is achieved in two ways, i.e., a way to reduce inputs without affecting targeted output and a way to improve output given the same number of inputs. This implies that if a producer’s objective is to maximize output, then DEA serves as the best tool to employ. Fundamentally, constant returns to scale (CRS) and variable returns to scale (VRS) are the two inherent models of DEA. Efficiency ratings and their measurement (scale efficiency) in this analysis were accounted for using CRS principles. Unlike VRS, where analyzed results vary due to inputs variation, results from CRS system always remain the same This facilitated smooth differentiation between the efficiency scores of CRS and VRS through their computational process. 

In addition, DEA helps in identifying the best DMUs’ production efficiency and measures the relative efficiency of others with reference to the best ones. The DMUs must demonstrate a sign of coexistence on the same frontier when using different combinations of inputs to produce different combinations of outputs. Hence, a farmer’s DMUs with output (Y) given (X) inputs are expected to churn out output (Y) employing the same input (X) with similar DMUs [27]. 

Therefore, applying the Tobit method, socioeconomic and institutional factors affecting fish farm production efficiency are regressed based on an output-oriented DEA VRS assumption in the final stage of the analysis, as specified [27]:

(1) $$\begin{array}{c} min_{\theta_{,}\lambda }\theta \;\\ subject\;to\\ -y_{i}+Y\lambda \geq 0\;\\ \theta x_{i}-X\lambda \geq 0\;\\ N1^{\prime }\lambda =1\\ \;\lambda \geq 0 \end{array}$$

where $N1^{\prime }$ is convexity constraint; θ is a scalar representing efficiency score of ith fish farm decision-making units; Y refers to the output matrix; and X represent the input matrix. λ refers to a (N×1) vector of a constant. Here, the condition λ≤1 is satisfied, with the value of 1 indicating a point on the frontier, i.e., production-efficient farm [31]. 

Subsequent to computing efficiency score through DEA approach, we adopted a two-tailed Tobit method to tease out the main sources of inefficiencies in the second stage. This is because efficiency rating falls between 0 and 1, i.e., a continuous variable. When it is censored at or below zero continuous values, the Tobit estimates are theoretically appropriate. 

Since the study aims to analyze the impact of household members’ NCDs on the production efficiency of fish farms, dealing with the potential endogeneity problem of the NCDs of household members variable in the Tobit model is essential. The NCDs of members become endogenous because of the mutual causality between household members with NCDs and efficiency. Thus, a household without any members with NCDs is more likely to increase efficiency, with greater output production compared with a household with more members with NCDs. In addition, an efficient household is likely to increase farm income through high productivity; hence, it is more likely to have access to healthcare services to reduce NCDs. Therefore, considering the nature of the dependent variable (censored and continuous) and the endogeneity issue, we employed the instrumental variable (IV) Tobit model to estimate the determinants of fish farm production efficiency [32]. We used the variable “health distance”, i.e., the distance from the respondent’s household to the nearest health facility (e.g., health center (traditional or non-traditional), clinic, or hospital) as an instrument. This instrument was chosen because it is expected to affect the household members’ NCDs status (treatment variable) but does not directly affect production efficiency (outcome variable). For example, if the distance is not too far, household members may be encouraged to utilize the nearest health facility [33]. Moreover, the 2017 report from the Ghana Health Ministry revealed that the inadequate healthcare infrastructure in most areas of the country contributes to the high prevalence of NCDs [34]. Particularly, the falsification test on the chosen IV in Appendix A (Table A1) shows that the selected IV is appropriate for the study. The falsification result implies that the IV only affects fish farm production efficiency through the household members’ NCDs.

The DEA VRS efficiency scores were regressed on several factors (endogenous and exogenous variables) in this model. The estimation of the household members’ NCDs status equation in the IV Tobit model can be expressed as follows:

(2) $$H_{i}=\gamma_{i}X_{i}+\theta_{i}Z_{i}+v_{i}$$

where $H_{i}$ refers to the number of NCDs members of household i; $X_{i}$ is a vector of explanatory variables; $\gamma_{i}$ and $\theta_{i}$ are the parameters to be estimated; $v_{i}$ is an error term that captures the unobserved effects; and $Z_{i}$ represents a vector of instrumental variable (IV), which is expected to affect household members’ health status but does not affect efficiency. The equation for the IV Tobit model regression is also specified as follows:

(3) $$Y_{\;i}^{*}=\alpha_{i}H_{i}+\beta_{i}X_{i}+\varepsilon_{i}\;Y_{i}=\max\left(0,{\;Y}_{\;i}^{*}\right)$$

where $Y_{\;i}^{*}$ is a latent variable; $Y_{i}$ measures the observed value of the production efficiency scores of farm i; $H_{i}$ is explained in Equation (2); $X_{i}$ is the vector of explanatory variables (Table 1); $\mu_{i}$ is an error term; $\alpha_{i}$ and $\beta_{i}$ are parameters to be estimated; and $\varepsilon_{i}$ represents the random disturbance term. Let us note that the regression model was tested for multicollinearity using a variance inflation factor (VIF). A recorded VIF result of 1.03 was generated. Since 1.03 is below the threshold of 10, we concluded that there is no serious multicollinearity problem between the explanatory variables used in the model [35].

## 3. Results and Discussions

### 3.1. Description Statistics

The average number of household members with NCDs in the study area is 2.93, of which the average number of male and female household members with NCDs were 3.14 and 2.73, respectively (Table 1). About 52% of the fish farms have access to credit, while 63% have access to extension services. The average age of the household heads in the sample is approximately 40 years. While household heads’ average years of formal education is approximately 11 years, the average years of fish farming (experience) is 12 years. About 66% of the household heads are part of a cooperative association, while 74% are engaged in off-farm activities. The average distance to the nearest health facility is 1.6 km, and the household size for the sampled group is 5.3. The average pond size, person-days of labor, feed cost, and other costs for the sampled group to achieve an average output of 6602.35 kg/ha are 0.49 ha, 12.3, GHS 1450, and GHS 1120, respectively. 

**Figure 1:**
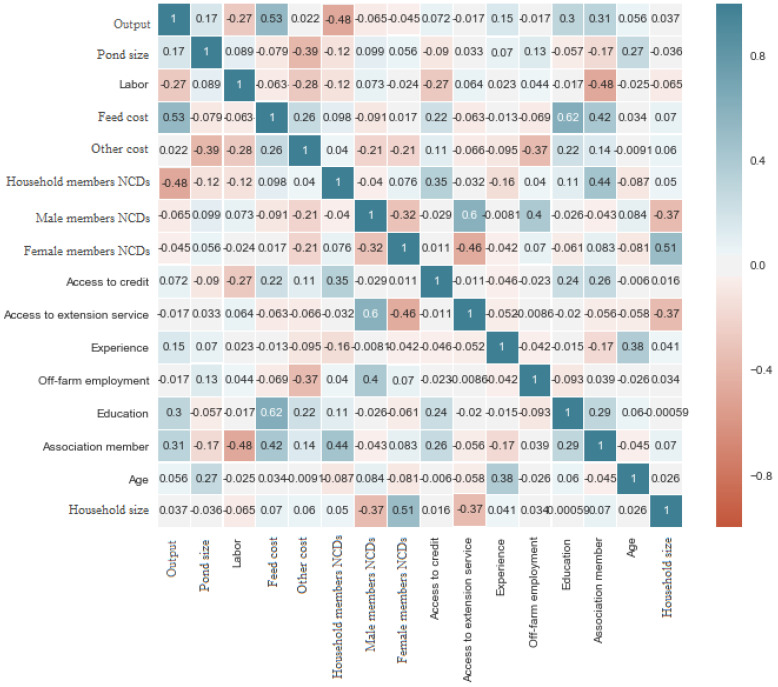
Heatmap for the matrix of Pearson’s correlation coefficients.

Additionally, given Figure 1 (heatmap), we developed a Pearson’s correlation coefficients matrix for the model variables. The colors in Figure 1 signify the values in the matrix. This implies that the lighter colors on the map represent the smaller absolute values of the correlation coefficient, and the reverse holds. Most of the areas are dominated by light colors from the heatmap, confirming our VIF result and suggesting the absence or insignificance of multicollinearity among the variables [9]. Figure 1 also shows that household members’ NCDs status and the quantity of the fish harvest (output) are negatively correlated. This implies that households with members with NCDs are less likely to increase their farm output. Nonetheless, to better grasp the quantitative correlation among the predictor variables, Figure 1 alone is insufficient because it does not account for other confounding variables when investigating the relationship between the expected and causal variables. 

### 3.2. Frequency Distribution of Technical Efficiency Scores 

The fish farms’ mean production efficiency level is about 79% for VRS, 41% for CRS, and 49% for SE (Table 2). These imply that for an efficient fish farm to maintain the same output level, the production costs should be reduced by approximately 21%, 59%, and 51% under VRS, CRS, and SE, respectively. While 9 fish farms were efficient under VRS out of the 131 total, only 3 each were efficient under CRS and SE, respectively. 

The households with a high number of members with NCDs are, on average, less efficient than their counterparts with a low number of members with NCDs (Table 3). Households who are members of an association are more efficient than those who are not members of an association. Similarly, fish farms run by farmers with more fishing experience are more efficient than those run by farmers with less fishing experience. Fish farm households with access to credit and extension services are more efficient than their counterparts. Fish farm households that do not engage in off-farm work are 4% more efficient than those involved in off-farm work. 

### 3.3. Determinants of Technical Efficiency 

To further assess the determinants of efficiency, we applied the IV Tobit model. Herein, the efficiency scores are regressed on the fish farmers and farm household characteristics. The endogenous Wald $X^{2}$ values of the model in Table 4 are all significant at a 1%level, indicating that the focal variable is endogenous and using IV Tobit to estimate the model is appropriate. The marginal effect results are based on the IV Tobit model. From Table 4, it is revealed that farm efficiencies are positively and significantly influenced by access to credit and extension services, cooperative membership, experience, and household size, while household members’ NCDs, age square, and off-farm employment negatively and significantly affect efficiency.

The results in Table 4 reveal that household members’ NCDs can reduce the production efficiency of fish farm households; thus, the relationship between household members’ NCDs and fish farm efficiency is negative and significant. The channeling of the time and funds required to increase production efficiency may be diverted to NCDs’ treatment or healthcare services. These findings have both similarities and differences with the previous literature. On the one hand, this study’s findings are similar to the findings of Dong [36] and Fink and Masiye [11], which suggested that farm productivity increases when farm managers’ and laborers’ health statuses are better. In addition, Lomari Apalia and Mutuku Kioko [37] revealed that HIV/AIDs and NCDs reduce fish farm production.

On the other hand, this study contradicts the studies of Currie and Madrian [38], which reported that in a low-income country, where agriculture is the only source of income for a household, health has no correlation with farm activities. The reason is that household members have no option other than to continue farming to improve their livelihood, even when they are unhealthy. However, productivity is improved by both the quality and quantity of laborers. Therefore, fish farm production efficiency is likely to fall if a larger share of household members are patients with NCDs [39].

Concerning the other control variables, the positive and significant marginal effect of access to credit implies that fish farm efficiency tends to increase if the farm household has access to credit facilities. These results are consistent with the findings of Chandio and Jiang [40] and Ankrah Twumasi [41], who argued that credit constraints deter farmers from purchasing the required inputs for production; hence, productivity is reduced through low efficiency. In addition, in this study, fish farm production efficiencies are improved by services from extension staff, a result consistent with [6]. Moreover, fish farm households that join a cooperative are far more efficient than their counterparts. The reason could be that association members help each other by providing financial support and training, which could enable them to achieve optimal satisfaction. This finding is consistent with the findings of [42,43,44], which reported that cooperative associations help farmers acquire new skills and ideas through discussions at a meeting. 

In addition, the positive and significant relationship between the years of farming experience and efficiency implies that farmers with many years of farming experience are more efficient than those with few years of farming experience. Better knowledge and skills are acquired as fish farmers’ years of farming experience increase. In addition, the household size’s positive relationship with farm efficiency implies that a larger family size tends to assist farm activities. Family members must, at times, serve as laborers during production. The result is consistent with the findings of Gül [45]. 

Moreover, off-farm employment and farming have a significant and negative relationship. The reason can be attributed to the fact that households with off-farm employment may have less time to concentrate on their own farm activities, which makes them inefficient. This result confirms the findings of Leng [46] and Ankrah Twumasi [47], which showed that off-farm activities inversely affect farm income. Finally, the coefficient of age square is negative and significant, indicating that farm efficiency reduces as farmers’ age increases. The strength to execute farm duties or intensify farming activities falls as farmers grow older. The study supports Mantey’s [48] findings. 

### 3.4. Heterogeneous Impact of Household Members’ NCDs on Production Efficiency by Gender Composition

This study’s heterogeneous results are presented in Table 5 by gender composition to gain further insights into the impact of the NCDs of household members on fish farm efficiency. In this section, the key variables, together with other control variables, are gradually added from model (1) to (3). In all the models, the Wald test values are significant at the 1% level, indicating that the null hypothesis (all variables are exogenous) is rejected; thus, employing the IV Tobit model is appropriate.

As shown in Table 5, the coefficients of the female and male members with NCDs have a negative sign. Thus, the farm efficiency of the female and male household members with NCDs is likely to reduce. However, the male members with NCDs coefficient’s absolute values are less than those of the female members with NCDs in models 1–3 but are insignificant in model 3, suggesting that production inefficiency is more profound among female members’ with NCDs than their male counterparts. In summary, the NCDs of female household members’ impact on fish farm production efficiency in the study areas is more robust compared with that of male members. The reason could be attributed to the household division of labor. In Africa, males have high financial responsibilities; therefore, they seek out addition jobs (off-farm jobs), which take up much of their farming-activities time [14]; therefore, females take care of the farm most of the time. 

### 3.5. Robustness Check Results

The introduction of the variable in a stepwise method in Table 4 could ensure the robustness of the estimated results. However, this study employed several identification strategies to ensure the robustness of the results in Table 6. First (model 1), the dependent variable (the fish farm efficiency scores) is replaced by the binary variable (1, if the farm efficiency score is above the efficiency sores median, and 0 otherwise), and the IV Probit model replaces the estimated method. Second (model 2), the study uses two-stage least squares (linear estimation) to replace the IV Tobit estimation method. As shown in Table 6, the relationship between the focal variable (household members with NCDs) and efficiency is significant at 1% and negative at levels (models 1 and 2), regardless of the estimation model’s changes. Thus, the results in Table 6 further confirm the estimated results in Table 4 and provide evidence that this research result is robust. 

## 4. Conclusions

The objective of this study was to explore the impact of the NCDs of household members on fish farms’ efficiencies using data from two regions (Bono East and Ashanti) in Ghana. From the study’s observations, we drew the following conclusions. First, the econometric approach suggests that fish farm production efficiencies are associated with the NCDs of household members’, credit accessibility, the availability and use of extension services, personal farming experience, being an association member, household size, age square, and access to off-farm work. Second, regarding the heterogeneous impact results, the impact of the NCDs of female household members on farm efficiency is paramount compared with that of males. Third, it was also observed that most of the fish farms are inefficient in their production. Specifically, out of the 131 fish farms, only 9 (6.9%), 3 (2.3%), and 3 (2.3%) are efficient under VRS, CRS, and SE, respectively. 

Given the above results, this study offered several implications. First, because the number of household members with NCDs has an adverse relationship with fish farms’ efficiencies, the government should provide these household members with the necessary medical care through the provision of subsidized health insurance, which can facilitate access to healthcare services. Moreover, NGOs and governments should encourage health literacy, i.e., organizing programs aimed at educating farmers on NCDs and their impact on agriculture. In addition, this study’s findings implied that there is an urgent need for policies that encourage fish farm households to coalesce under unions and cooperative organizations. This recommendation is grounded in the fact that cooperative members were more efficient in their production. Arguably, exchanging ideas and skills within such groups contributed to upscaling members’ skills and knowledge, thereby enhancing their production efficiency. Further, considering the significant positive association among credit accessibility, the provision of extension services, and fish farmers’ production efficiency, policymakers and the major stakeholders should prioritize the provision of such facilities and services to fish farm households to boost their production capacity. Finally, the government and other stakeholders promoting fish farming by females is essential because, compared to males, healthy females have a greater impact on fish farms’ efficiencies. 

Furthermore, in this study, several limitations can be addressed by future researchers. First, the study focused on only two regions in Ghana due to financial constraints. Future researchers can consider a larger sample size, probably the nation as a whole. Second, this study focused on NCDs for this quantitative analysis. Future researchers can consider other health statuses, such as the households’ psychological health and its impact of production. Finally, the study focused on earthen pond fish farm households; future researchers can further explore households using other aquaculture systems, such as cage farms. 

## Figures and Tables

**Table 1:** Socioeconomic, demographic, and other characteristics of the respondents.

Variables	Description	Mean	Std. Dev.
Input/output variables			
Output	Total output per hectare in the past 12 months	6602.35	4701.10
Pond size	Total fish farm land in hectares (Ha)	0.49	0.78
Labor	Person-days/Ha	12.32	5.46
Feed cost	Feed cost (GHS 1000/capita)	1.45	2.85
Other costs	Total cost of other inputs (GHS 1000/capita)	1.12	2.71
Characteristics			
Household NCDs	Number of household members with NCDs	2.93	1.65
Male NCDs	Number of male household members with NCDs	3.14	27.84
Female NCDs	Number of female household members with NCDs	2.73	28.87
Access to credit	1, if the household has access to credit, and 0 otherwise	0.52	0.46
Access to extension service	1, if the household has access to extension services, and 0 otherwise	0.63	0.48
Off-farm employment	1, if the household have off-farm employment, and 0 otherwise	0.74	0.51
Education	Respondent’s years of formal education	10.90	4.51
Cooperative member	1, if the respondent is a member of a cooperative, and 0 otherwise	0.66	0.47
Age	Respondent’s age	40.13	7.72
Experience	Years of farming experience	11.75	2.43
Household size	Total household size	5.29	1.13
Health distance	Distance from the respondent’s resident to the nearest health facility (kilometers)	1.53	0.48

Source: Survey results, 2018.

**Table 2:** Frequency distribution of technical efficiency scores.

Efficiency Score	VRS	CRS	SE
0.00–0.09	0	4	0
0.10–0.19	0	44	14
0.20–0.29	7	23	22
0.30–0.39	12	19	29
0.40–0.49	23	15	23
0.50–0.59	21	6	19
0.60–0.69	15	2	5
0.70–0.79	24	7	8
0.80–0.89	13	5	7
0.90–0.99	7	3	1
1	9	3	3
Total DMUs	131	131	131
Min.	0.286	0.071	0.186
Max.	1	1	1
Mean	0.791	0.413	0.493
SD	0.115	0.273	0.314

Source: Survey results, 2018.

**Table 3:** Summary results of the VRS technical efficiency scores by household characteristics.

Characteristics	Mean	Std. Dev	*p*-Value
Number of household members with NCDs			
High number of NCDs	0.745	0.043	0.0007
Low number of NCDs	0.837	0.162	
Cooperative member			
Member	0.805	0.103	0.0084
Non-member	0.777	0.176	
Years of farming experience			
Low experience	0.769	0.108	0.0022
High experience	0.813	0.090	
Access to credit			
Have access to credit	0.819	0.147	0.0004
Do not have access to credit	0.763	0.103	
Access to extension services			
Have access to extension services	0.799	0.122	0.0461
Do not have access to extension services	0.783	0.034	
Off-farm employment			
Access to off-farm work	0.771	0.072	0.0012
Non-access to off-farm work	0.811	0.110	

Source: Survey results, 2018. Note: We divided the sample into two groups based on farming experience level and number of household member with NCDs, with the median in the sample as the breakpoint.

**Table 4:** Tobit model results of the determinants of fish farms’ efficiency.

		VRS	
Variables	Tobit Model	IV Tobit	Marginal effect
Household members with NCDs	−0.0145 (0.0135) *	−0.2738 (0.0248)	−0.2101 (0.0226) **
Access to credit	0.0470 (0.0275) **	0.0253 (0.0251)	0.0127 (0.0151) **
Access to extension services	0.0511 (0.0021) *	0.0239 (0.0158)	0.0159 (0.0045) *
Off-farm employment	−0.0241 (0.0120) *	−0.0756 (0.0237)	−0.0693 (0.0163) *
Education	0.0321 (0.0698)	0.0362 (0.0327)	0.0227 (0.0145)
Cooperative member	0.0221 (0.0162)	0.0451 (0.0313)	0.0341 (0.0182) *
Age	0.0077 (0.0055)	0.0094 (0.0021)	0.0066 (0.0038)
Age^2^	−0.0001 (0.0000) *	−0.0001 (0.0000)	−0.0001 (0.0000)
Experience	0.0479 (0.0126) ***	0.0261 (0.0172)	0.0210 (0.0143) ***
Household size	0.0063 (0.0043)	0.0077 (0.0042)	0.0006 (0.0009) *
Constant	1.1407 (0.0212)	1.1447 (2.1012)	
Regions	Yes	Yes	Yes
Instrumental variables	No	Yes	Yes
Endogenous Wald $X^{2}$		67.31 ***	67.31 ***
Observation	131	131	131

Source: Survey results, 2018. Note: *** *p* < 0.01, ** *p* < 0.05, * *p* < 0.1. Standard errors in parentheses.

**Table 5:** The estimated results for the impact of household health on fish farm efficiency by gender composition.

Variables	Model 1	Model 2	Model 3
Female members with NCDs	−0.0677 (0.1036) ***	−0.1004 (0.1042) ***	−0.1851 (0.1244) ***
Male members with NCDs	−0.0251 (0.0261) *	−0.0981 (0.0261) *	−0.0890 (0.0249)
Control variables	No	No	Yes
Regional dummies	No	Yes	Yes
Instrumental variables	Yes	Yes	Yes
Wald $X^{2}$	76.66 ***	76.07 ***	77.34 ***

Source: Survey results, 2018. Note: *** *p* < 0.01, * *p* < 0.1. Standard errors in parentheses.

**Table 6:** Estimated results of robustness check.

Variables	Model 1 (IV Probit)	Model 2 (2SLS)
Household members with NCDs	−0.1513 (0.0774) ***	−0.1901 (82.0103) ***
Control variables	Yes	Yes
Regional dummies	Yes	Yes
Instrumental variables	Yes	Yes
Wald $X^{2}$	63.27 ***	65.17 ***
Observation	131	131

Source: Survey results, 2018. Note: *** *p* < 0.01. Standard errors in parentheses.

## Data Availability

The data used for the study are private but can be made available upon reasonable request.

## Appendix A

**Table A1:** Falsification analysis to test the selected IV’s validity.

IV	Outcome Variable	Statistics
Health distance	Household members’ NCDs	$X^{2}$= 43.34 **; *p*-value = 0.027
Health distance	Fish farm efficiency	F-value= 0.56; *p*-value = 0.258

Source: Survey results, 2018. Note: ** *p* < 0.05.
